# One Health Antimicrobial Resistance in Qatar: A Comprehensive Systematic Review and Meta-Analysis of Animal, Food, and Environmental Reservoirs

**DOI:** 10.3390/antibiotics14121219

**Published:** 2025-12-03

**Authors:** Lubna I. Abu-Rub, Ristha Kamar, Cut Salsabila Fatin, Susu M. Zughaier, Nahla O. Eltai

**Affiliations:** 1 Microbiology Department, Biomedical Research Center, Qu Health Sector, Qatar University, Doha P.O. Box 2713, Qatar; lubna.ali@qu.edu.qa (L.I.A.-R.); ristha.k@qu.edu.qa (R.K.); cf1805126@student.qu.edu.qa (C.S.F.); 2 College of Medicine, Qatar University, Doha P.O. Box 2713, Qatar; szughaier@qu.edu.qa

**Keywords:** AMR prevalence, resistance genes, One Health, antibiotics, surveillance, Qatar

## Abstract

**Background**: Antimicrobial resistance (AMR) is a global threat that extends beyond clinical settings, impacting animals, food, and the environment. To the best of our knowledge, this review presents the first systematic evaluation of AMR and antimicrobial resistance genes (ARGs) in non-human sources in Qatar, using a One Health framework. **Methods**: Following PRISMA 2020 guidelines, we searched five major databases: PubMed, Scopus, Web of Science, Embase, Google Scholar (only 3 pages) and QRDI, without date restrictions for studies on AMR and ARGs in animals, food, and environmental sources in Qatar. Only primary studies from Qatar reporting phenotypic or genotypic AMR/ARG data in animals, food, or the environment were included; all human-focused, non-Qatar, or non-primary research were excluded. Eligible studies were screened and analyzed using GraphPad Prism 10.4 and StatsDirect, applying random- or fixed-effects models based on heterogeneity and assessed for quality using the JBI checklist for prevalence. **Results**: Fifteen eligible studies published up to 2025 were included. *Escherichia coli* was the most frequently reported organism. High resistance rates were observed in the Access group antibiotics, such as ampicillin (0.50; 95% CI: 0.47–0.53) and tetracycline (0.50; 95% CI: 0.45–0.55), as well as in the Watch group antibiotics, including ciprofloxacin (0.40; 95% CI: 0.36–0.44) and fosfomycin (0.26; 95% CI: 0.20–0.32). Resistance to Reserve group antibiotics was comparatively lower, with pooled estimates of 0.14 (95% CI: 0.08–0.20) for colistin and 0.11 (95% CI: 0.05–0.25) for carbapenems, though lower, remains concerning. The overall pooled estimate for multidrug resistance (MDR) was 0.56 (95% CI: 0.36–0.72), and poultry was identified as the main reservoir, particularly to Critically Important Antimicrobials (CIAs). ARGs, including *bla*_CTX-M_, *bla*_TEM_, *mcr-1*, and *qnr*, were detected across all sectors, with wastewater showing a notable ARG burden. Data on other livestock species remain limited. Limitations include a few studies, variable quality, and inconsistent methods affecting comparability and precision. **Conclusions:** This review highlights significant AMR and ARG prevalence in non-human sources in Qatar and underscores the urgent need for a national One Health surveillance strategy incorporating WHO AWaRe and CIA frameworks to address this escalating public health threat.

## 1. Introduction

AMR is a major global threat to public health and development [1]. The continuous rise and spread of AMR in bacteria is driving medicine toward a post-antibiotic era [2]. In 2019, an estimated 1.27 million deaths were directly attributable to antimicrobial resistance (AMR), with a further 4.95 million deaths associated with resistant infections globally [3]. Projections suggest that AMR could cause up to 10 million deaths annually by 2050, highlighting a critical threat to global public health [3]. Without adequate action, bacterial pathogens may become significantly more dangerous [4]. Consequently, AMR surveillance is crucial for guiding policies and strategies. In response, the World Health Organization (WHO) launched the Global AMR and Use Surveillance System (GLASS) in 2015 to standardize and enhance global monitoring efforts [5]. Qatar was enrolled in the GLASS program in 2019 and is actively monitoring AMR through national initiatives, such as the Tracking AMR Country Self-Assessment Survey (TrACSS) [5]. This initiative highlights improvements in animal health but identifies gaps in plant production, training, and surveillance across the animal health, agriculture, and food sectors [6,7]. Addressing AMR requires a One Health approach that integrates data from clinical, agricultural, and environmental sectors to model transmission and assess control strategies [8]. Despite challenges such as data gaps and biases across animal, food, and environmental sources, methodological inconsistencies, and the inherent complexity of AMR systems, these efforts emphasise the need for a comprehensive cross-sectoral AMR response [9]. The WHO’s One Health approach strategy focuses on five pillars: increasing AMR awareness, strengthening surveillance, preventing infections, optimizing antimicrobial use, and ensuring sustainable investment in accessible medical solutions [10]. In the Eastern Mediterranean Region, including the Gulf countries, progress toward One Health implementation has been achieved; however, challenges such as weak governance, limited surveillance integration, and insufficient funding still constrain efforts to combat AMR effectively [11]. The Gulf Cooperation Council Center for Infection Control (GCC-IC) developed a strategic plan to understand AMR, preserve antibiotics (Abs), detect resistance, limit pathogen spread, and promote AMR research, while tackling challenges such as leadership support and performance indicators [2]. Bansal et al. (2023) noted institutional and policy gaps in Qatar that impede full One Health operation, expected the One Health Framework (OHF) to enhance multi-sectoral coordination, monitoring, and prevention across human, animal, and environmental sectors [12].

A systematic review of Qatar’s human populations found AMR prevalence comparable to global trends, while also highlighting higher resistance rates than in other high-income countries, underscoring the need for improved public health responses and broader epidemiological research [13]. Building on this, the current review examines AMR in non-human sources in Qatar, including animals, the environment, and food. This review systematically synthesizes published data to assess the prevalence and patterns of AMR across bacterial species and antimicrobial agents in animals, food, and the environment in Qatar, and to identify associated risk factors. In alignment with WHO recommendations, country-specific AMR mapping represents a crucial preliminary step before meaningful regional or global comparisons can be conducted. This study was undertaken at the request of the national authorities to inform the Qatar National Action Plan for AMR (2022–2030) and to fill a critical evidence gap identified by international and national stakeholders. The findings underscore the urgent need to strengthen surveillance systems, infection prevention and control measures, and antimicrobial stewardship programs to mitigate the growing AMR threat within the One Health framework in Qatar.

## 2. Results

### 2.1. Outcome of Study Identification Process

**Figure 1:**
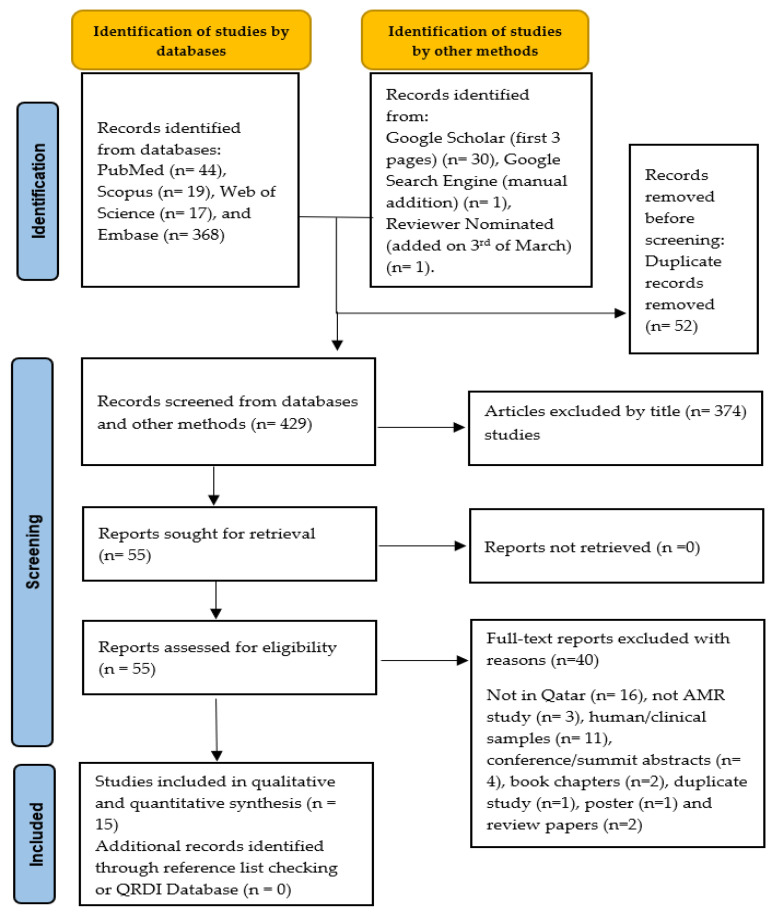
PRISMA schematic selection process of the included studies at each stage of the screening process.

The literature search retrieved 481 articles from five databases, including PubMed, Scopus, Web of Science, Embase, and Google Scholar (first 3 pages). After removing duplicates and screening titles/abstracts, 55 articles were selected for retrieval. During the full-text screening, 40 articles were excluded after applying the inclusion and exclusion criteria, resulting in 15 studies being included in the systematic review (Figure 1).

### 2.2. Study Characteristics

**Figure 2:**
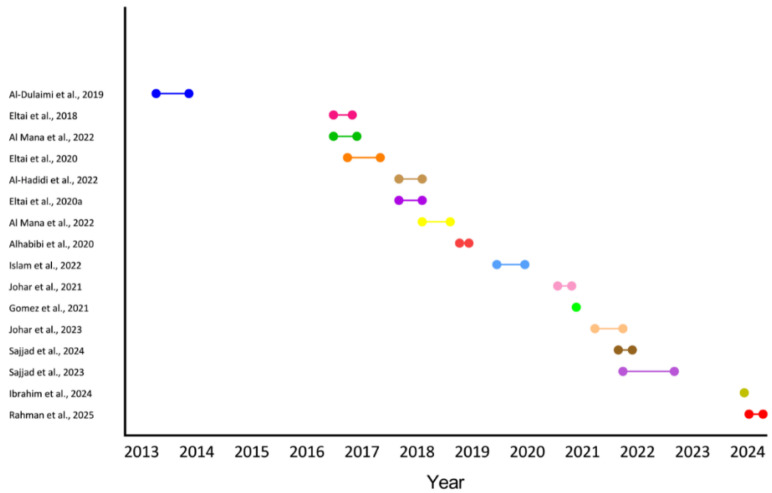
Sampling periods of included studies. Each horizontal line represents the timeframe during which samples were collected for a given study [14,15,16,17,19,20,21,22,23,24,25,26,28].

Among the included studies [14,15,16,17,18,19,20,21,22,23,24,25,26,27,28] (Table 1), 40% (n = 6) investigated AMR in food sources with a strong emphasis on poultry products [14,15,16,17,18,19]. Meanwhile, studies focused on live animals were 33.3% (n = 5) [14,21,22,23,24], and the environmental studies were 26.7% (n = 4) [25,26,27,28]. All included studies were cross-sectional in design, with sample sizes ranging from a minimum of 12 to a maximum of 300 [22,28]. The sampling periods across the included studies spanned over a decade, reflecting a wide temporal range in AMR surveillance. Most studies collected samples between 2013 and 2024, with a noticeable concentration during the years 2016 to 2022. The earliest sampling occurred from July 2013 to February 2014, while the most recent was between February and May 2024 (Figure 2 and Table 1). 

### 2.3. Risk of Bias Assessment

**Figure 3:**
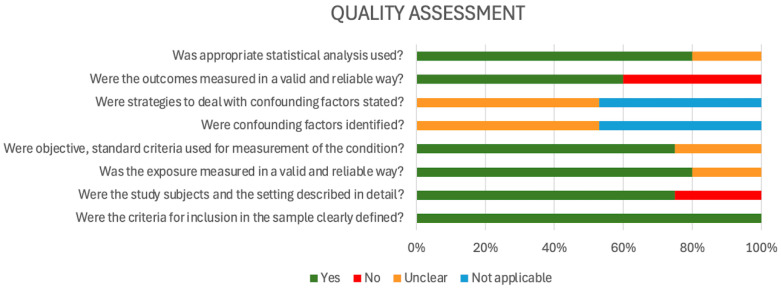
Quality assessment (risk of bias) using the JBI tool (2017).

For the quality assessment (risk of bias) using the JBI tool [29], most studies exhibited a low risk of bias in several critical domains. Specifically, 100% of studies clearly defined their inclusion criteria and used appropriate statistical analyses. A high proportion also measured exposures (92.9%) and outcomes (78.6%) validly and reliably. Additionally, objective and standardized measurement criteria were used in 78.6% of studies, suggesting methodological rigor in data collection processes. However, areas of concern emerged in the assessment of confounding control. Only a minority of studies adequately identified confounding factors or stated strategies to address them. Instead, the majority were rated as either “unclear” or “not applicable” in these domains, indicating insufficient reporting or a lack of methodological measures to reduce confounding bias. This raises concerns about potential distortion of study findings and may affect the internal validity of reported AMR estimates. Furthermore, although most studies described their settings and participants, about 21.4% lacked sufficient detail, and a similar proportion showed limitations in outcome measurement reliability. These gaps suggest potential risks in the reproducibility and contextual interpretation of findings, particularly in observational AMR studies (Figure 3).

### 2.4. Bacterial Isolates, Tested Abs Panel, and ARG’s Distribution

Different bacterial species were isolated across the “animals”, “food products”, and “environmental” sectors. *Escherichia* (*E*) *coli* was the most frequently studied species (66.7%), followed by *Pseudomonas* (*P*) *aeruginosa* (26.7%). *Klebsiella* (*K*) *pneumoniae*, *Salmonella* spp., and *Proteus* spp. were each reported in 20% of studies, and *Acinetobacter* spp. was in 13.3% of the studies (Table S2) [14,15,16,17,18,19,20,21,22,23,24,25,26,27,28].

For the antimicrobial susceptibility testing, 14 out of 15 studies conducted the AST. Among these, 9 utilized the Kirby-Bauer disk diffusion test, whereas six used the E-test method. One study used the VITEK-2 system, and another used the PHOENIX system. These studies examined resistance patterns to 55 different antibiotics. The most commonly tested antibiotics were ampicillin (86.7%), ciprofloxacin (80%), trimethoprim-sulfamethoxazole (80%), and amoxicillin-clavulanic acid (80%), followed by tetracycline (66.7%), meropenem (60%), ertapenem (60%), ceftriaxone (60%), fosfomycin (60%), and nitrofurantoin (60%) (Table S3) [14,15,16,17,18,19,20,21,22,23,24,25,26,27].

A wide range of ARGs were identified across the reviewed studies conducted in Qatar. The *bla*_CTX-M_ gene was consistently reported in multiple studies, including those by Eltai et al. (2020) [14], Rahman et al. (2025) [24], and Ibrahim et al. (2024) [27]. *bla*_TEM_ and *bla*_SHV_ were also frequently identified, with detections by Eltai et al. (2020) [14], Gomez et al. (2021) [18], and Ibrahim et al. (2024) [27]. A specific variant, *bla*_CTX-M-G2_*,* was exclusively reported by Eltai et al. (2020) [21]. The colistin resistance gene *mcr-1* was detected in several studies, including those by Eltai et al. (2020) [14], Al Mana et al. (2022) [17], Eltai et al. (2018) [20], and Alhababi et al. (2020) [22], while Ibrahim et al. (2024) [27] reported a broader *mcr* gene category. Other β-lactamase genes included *bla*_KPC_, *bla*_NDM_, *bla*_OXA_, *bla*_VIM_, *bla*_IMP_, and *bla*_VEB_, as reported in studies by Ibrahim et al. (2024) [27] and Johar et al. (2023) [28], who also identified additional variants such as *bla*_GES_, *bla*_VIM_-_1_, and *bla*_OXA_ variants. Aminoglycoside and macrolide resistance genes such as *aadA*, *aadA*1, *aadA*, *ermB*, and mefA were also reported by Gomez et al. (2021) [18] and Johar et al. (2023) [28]. Fluoroquinolone resistance determinants such as *qnr*, *qnrB-1*, and *qnrS* were identified by Ibrahim et al. (2024) [27] and Johar et al. (2023) [28]. Tetracycline resistance genes *tetA* and *tetB* were documented in studies by Rahman et al. (2025) [24] and Johar et al. (2023) [28], respectively. Efflux pump and multidrug resistance genes were extensively documented in Rahman et al. (2025) [24], including *acrS*, *soxR* (with mutation), *emrA*, *acrAB-tolC* (with *acrR* and *marR* mutations), *fabI* (mutations), *qacG*, *mdtE*, *emrB*, *mdtF*, and *marA*. Additionally, genes related to shiga toxin and *E. coli* virulence—*stx* and *eae*—were identified in Gomez et al. (2021) [18], while the *AAc(6’)-1b-cr* plasmid-mediated quinolone resistance gene was found in Johar et al. (2023) [28] (Table S4) [14,16,17,18,20,22,24,27,28].

### 2.5. Meta-Analysis Results

#### 2.5.1. Pooled AMR Prevalence of the Most Commonly Studied Antibiotics

**Figure 4:**
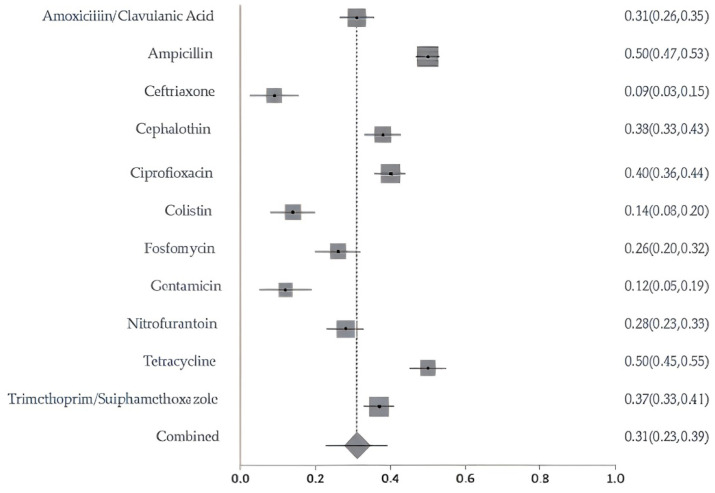
Forest plot of pooled resistance rates for selected antibiotics with 95% confidence intervals. X-axis shows the correlation coefficient (r); Y-axis shows the antibiotic studied. The squares represent the resistance rate for each study, with horizontal lines depicting the 95% confidence intervals. The diamond represents the overall pooled resistance rate.

A meta-analysis of resistance to 11 commonly used antibiotics revealed pooled prevalence estimates ranging from 9% for ceftriaxone and 14% for colistin, up to 50% for tetracycline and ampicillin. Cephalothin and ciprofloxacin also showed elevated resistance levels of 38% and 40%, respectively. The overall pooled resistance across all antibiotics was estimated at 31% (95% CI: 0.23–0.39) (Figure 4). The meta-analysis of resistance to key antibiotics showed significant heterogeneity across studies (Cochran’s Q = 301.24, df = 10, *p* < 0.0001). The estimated between-study variance (τ^2^) was 0.02266. A high level of inconsistency was observed, with an I^2^ of 96.7% (95% CI: 95.8–97.3%), indicating substantial variability not attributable to chance alone. Egger’s regression test for publication bias was not statistically significant (*p* = 0.0254), indicating no strong evidence of funnel plot asymmetry. The sensitivity of pooled AMR prevalence was stable. The I^2^ and Cochran’s Q statistics remained largely consistent across iterations, suggesting that no single study exerted a disproportionate influence on the overall findings. These results confirm the robustness and reliability of the pooled prevalence estimates.

#### 2.5.2. Resistance Patterns and Meta-Analysis by Source: Animals, Food Products, and Environmental Samples

**Figure 5:**
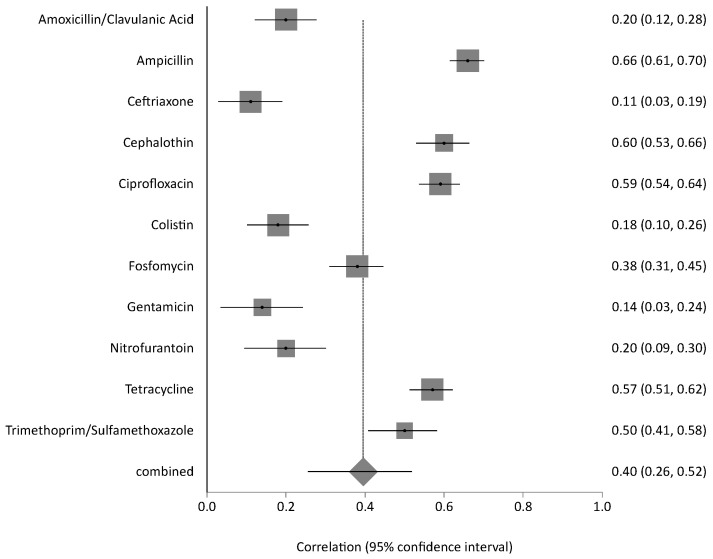
Forest plot of pooled resistance rates from food-derived bacterial isolates with 95% confidence intervals. X-axis shows the correlation coefficient (r); Y-axis shows the antibiotic studied. The squares represent the resistance rate for each study, with horizontal lines depicting the 95% confidence intervals. The diamond represents the overall pooled resistance rate.

Bacterial isolates from livestock, poultry, and wild animals were reported in 5 studies and exhibited high resistance to several antibiotics. Chickens showed the highest resistance, with ampicillin at 72%, trimethoprim/sulfamethoxazole at 63%, and ciprofloxacin at 40%. Rodents and pigeons also demonstrated elevated resistance, particularly to tetracycline at 67% and 64%, respectively; ampicillin at 56% and 55%; and trimethoprim-sulfamethoxazole at 35% and 49%. Rodents additionally showed high resistance to nitrofurantoin at 61%. Sheep isolates exhibited high resistance to ciprofloxacin (69%), nitrofurantoin (47%), trimethoprim-sulfamethoxazole (46%), and cephalothin (43%). In contrast, isolates from camels, cattle, and oryx exhibited relatively low resistance levels across all antibiotics tested (Figure S1). The pooled analysis of AMR in animal-derived samples revealed significant heterogeneity (Q = 70.10, df = 7, *p* < 0.0001). The estimated between-study variance was 0.01391, with an I^2^ value of 90% (95% CI: 82.9–93.3%), indicating high inconsistency among the included studies. (Figure 5). The overall resistance across animals’ AMR category was estimated at 26% (95% CI: 0.17–0.33). The contributing animal studies were small to moderate in size, with low-to-moderate risk of bias. The sensitivity of pooled AMR rates from animal-derived bacterial isolates was stable. The I^2^ and Cochran’s Q statistics remained consistent, confirming the robustness and reliability of the prevalence.

**Figure 6:**
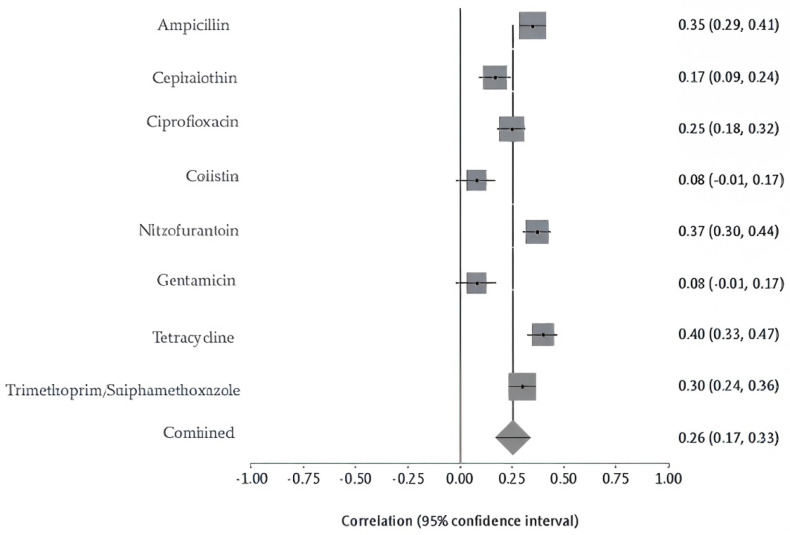
Forest plot of pooled resistance rates from animal-derived bacterial isolates with 95% confidence intervals. The X-axis shows the correlation coefficient (r); the Y-axis shows the antibiotic studied. The squares represent the resistance rate for each study, with horizontal lines depicting the 95% confidence intervals. The diamond represents the overall pooled resistance rate.

Moreover, among food products (animal-derived) samples, chicken isolates had the highest resistance rates, including tetracycline at 66%, ampicillin at 65%, cephalothin at 62%, ciprofloxacin at 59%, and trimethoprim-sulfamethoxazole at 51%. Seafood isolates demonstrated a high level of resistance to penicillin (91%), ampicillin (74%), vancomycin (65%), clindamycin (61%), and cephalothin (57%). In comparison, meat-derived isolates showed high resistance to erythromycin at 91% and penicillin at 88%, with low resistance to streptomycin at 25% and tetracycline at 21%. Resistance to amoxicillin-clavulanic acid, ceftriaxone, colistin, gentamicin, and nitrofurantoin remained low across all food sources (Figure S2). Meta-analysis for this category displayed marked heterogeneity (Q = 347.40, df = 10, *p* < 0.0001), with a between-study variance of 0.06812. The inconsistency across studies was high, as reflected by an I^2^ of 97.1% (95% CI: 96.4–97.6%), pointing to significant differences in findings across food-related sources (Figure 6). The overall pooled resistance across all antibiotics was estimated at 40% (95% CI: 0.26–0.52). The contributing food-related studies were moderate in size and exhibited low-to-moderate risk of bias. The sensitivity of pooled AMR rates from food-derived bacterial isolates was stable. The I^2^ and Cochran’s Q statistics remained consistent, confirming the robustness and reliability of the prevalence.

**Figure 7:**
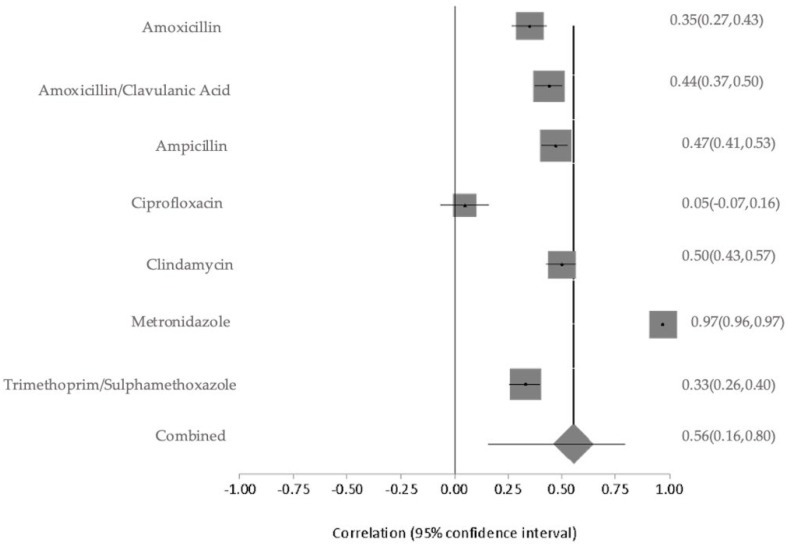
Forest plot of pooled resistance rates from environmental bacterial isolates with 95% confidence intervals. The X-axis shows the correlation coefficient (r); the Y-axis shows the antibiotic studied. The squares represent the resistance rate for each study, with horizontal lines depicting the 95% confidence intervals. The diamond represents the overall pooled resistance rate.

Environmental samples collected from public restrooms and the surrounding air demonstrated different resistance levels. Isolates from public restrooms exhibited slightly higher AMR, with ampicillin at 63%, amoxicillin-clavulanic acid at 50%, and lower resistance to cephalothin at 38%. In contrast, ambient air isolates showed very high resistance to metronidazole at 98% and lower resistance levels to clindamycin at 50%, trimethoprim-sulfamethoxazole at 44%, amoxicillin-clavulanic acid at 42%, and ampicillin at 39% (Figure S3). The meta-analysis of the environmental sources exhibited extremely high heterogeneity in AMR prevalence (Q = 1178.48, df = 6, *p* < 0.0001). The between-study variance was the highest among all categories at 0.39463, and the I^2^ was 99.5% (95% CI: 99.4–99.5%), suggesting nearly all variability was due to differences across studies rather than random error. This extreme variability is likely due to the broad range of environmental matrices examined (e.g., air, public restrooms), variable exposure conditions, and inconsistent detection methodologies (Figure 7). The overall pooled resistance across all antibiotics was estimated at 56% (95% CI: 0.16–0.80). The contributing environmental studies varied in sample type and size, with a moderate risk of bias due to methodological differences. The sensitivity of pooled AMR rates from environmental bacterial isolates was stable. The I^2^ and Cochran’s Q statistics remained consistent, confirming the robustness and reliability of the prevalence.

#### 2.5.3. Resistance Patterns and Meta-Analysis of Critically Important Antimicrobials

**Figure 8:**
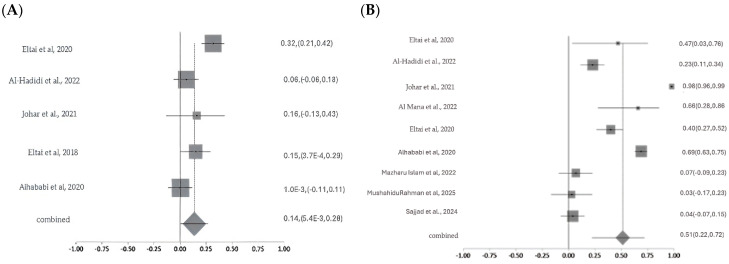

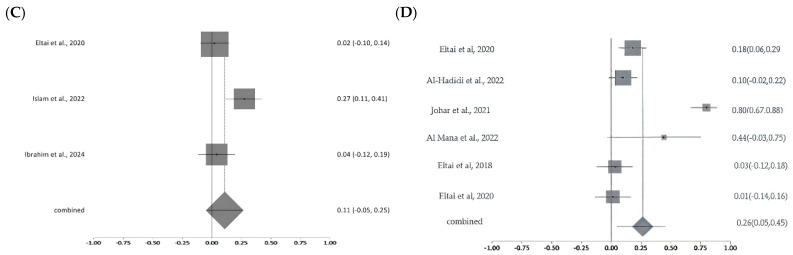
Forest plots showing pooled prevalence of AMR across included studies for selected critically important antibiotics. Forest plots of pooled AMR prevalence for Critically Important Antimicrobials (CIA): (**A**). Colistin, (**B**). Ciprofloxacin, (**C**). Carbapenems, and (**D**). Fosfomycin. The Y-axis shows the studies investigated, and the X-axis shows the correlation coefficient (r). The squares represent the resistance rate for each study, with horizontal lines depicting the 95% confidence intervals. The diamond represents the overall pooled resistance rate [14,15,16,17,20,21,22,23,24,26,27].

Meta-analysis of colistin resistance revealed a pooled prevalence of 14% with moderate-to-high heterogeneity (Cochran’s Q = 17.10, *p* = 0.0018; I^2^ = 76.6%), indicating variability likely due to sample sources, geography, or detection methods. Ciprofloxacin resistance, analyzed across nine studies, showed a higher pooled prevalence of 51% with substantial heterogeneity (Q = 295.50, df = 8, *p* < 0.0001; I^2^ = 97.3%). For carbapenems, pooled resistance was 11% based on three studies, with moderate heterogeneity (Q =6.65, df = 2, *p* = 0.036; I^2^ = 69.9%). Fosfomycin resistance estimates ranged widely (1% to >80%) across studies, with a pooled prevalence of 26% and significant heterogeneity (Q = 47.64, *p* < 0.0001; I^2^ = 89.5%), reflecting differences in study design, isolate origin, and methodologies (Figure 8A–D). The studies were moderate in scale and quality, with variability in methods and sample sources contributing to a moderate-to-high risk of bias. Sensitivity analyses were conducted for colistin, ciprofloxacin, carbapenem, and fosfomycin to assess the robustness of pooled antimicrobial resistance (AMR) estimates and identify sources of heterogeneity. For colistin, heterogeneity was high across most iterations (I^2^ = 70.4–81.8%; Cochran’s Q = 10–17), but removal of a single study markedly reduced heterogeneity (I^2^ = 0%; Q = 2.5), suggesting that this study was the primary driver of variability; however, the pooled resistance estimate remained stable, confirming the robustness of the findings. In the case of ciprofloxacin, sensitivity analysis across nine studies revealed minimal variation in I^2^, Cochran’s Q, and Chi-square values (<3%), indicating that no individual study significantly influenced the overall estimate and supporting the reliability of the pooled results. For carbapenems, moderate heterogeneity was observed (I^2^ = 55–76%), but it dropped to 0–0.4% upon exclusion of certain studies, with corresponding decreases in Q and Chi-square values (from 4.5 to 0–1), implicating one or two studies in the observed variability; yet, the pooled resistance estimate remained consistent. Fosfomycin analysis showed high heterogeneity (I^2^ = 89–91%; Q = 43–47), which declined substantially (I^2^ = 34%; Q = 6) when one study was excluded, indicating that this study heavily contributed to variability. While this suggests some instability, the overall estimate showed moderate robustness.

#### 2.5.4. Pooled AMR Prevalence of the Most Commonly Studied Non-CIAs

**Figure 9:**
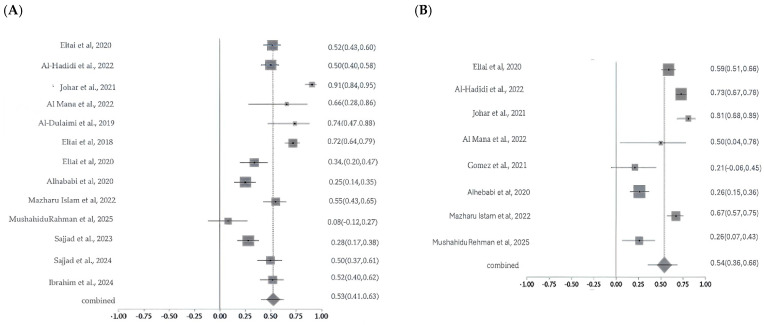
Forest plot showing ampicillin (**A**) and tetracycline (**B**) resistance across the studies with 95% confidence intervals. The Y-axis shows the studies investigated, and X-axis shows the correlation coefficient (r). The squares represent the resistance rate for each study, with horizontal lines depicting the 95% confidence intervals. The diamond represents the overall pooled resistance rate [14,15,16,17,18,19,20,21,22,23,24,25,26,27].

Resistance to ampicillin and tetracycline was moderately high across the included studies, with pooled prevalence estimates of 53% and 54% respectively. Significant heterogeneity was observed for both antibiotics (ampicillin: Cochran’s Q = 137.23, *p* < 0.0001, I^2^ = 91.3%; tetracycline: Cochran’s Q = 100.94, *p* < 0.0001, I^2^ = 93.1%), indicating substantial variability likely attributable to differences in isolate sources, geographic regions, and methodological approaches (Figure 9A,B). The studies assessing non-CIA antibiotic resistance were of small to moderate scale and generally exhibited low to moderate methodological limitations. Egger’s regression test for ampicillin and tetracycline was not statistically significant (*p* > 0.05), indicating no strong evidence of funnel plot asymmetry. Sensitivity analyses for ampicillin and tetracycline resistance further supported the robustness of the pooled estimates despite varying levels of heterogeneity. For ampicillin, I^2^ values consistently exceeded 85%, and Cochran’s Q remained stable across iterations, indicating substantial but consistent heterogeneity; importantly, no single study significantly influenced the pooled effect estimate, confirming the stability and reliability of the findings. Similarly, for tetracycline, I^2^ values remained consistent throughout the analysis, while exclusion of one study led to a notable reduction in Cochran’s Q (from approximately 83–95 to 45), suggesting that this study contributed disproportionately to heterogeneity. Despite this, the pooled resistance estimate remained stable, reinforcing the robustness of the overall results.

#### 2.5.5. Prevalence and Characterization of ESBL Production

**Figure 10:**
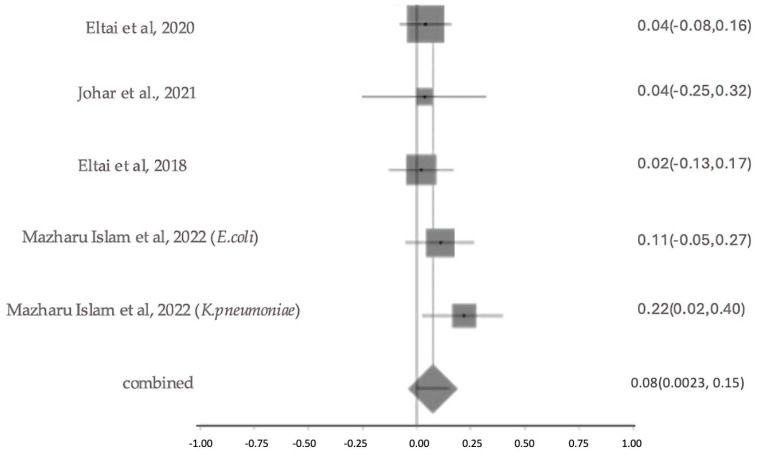
The forest plot shows ESBL Resistance Patterns across studies with 95% confidence intervals. The Y-axis shows the studies investigated, and the X-axis shows the correlation coefficient (r). The squares represent the resistance rate for each study, with horizontal lines depicting the 95% confidence intervals. The diamond represents the overall pooled resistance rate [14,16,20,23].

The prevalence of ESBL-producing organisms varied considerably across the studies. Eltai et al. (2020) [14] reported a confirmed ESBL rate of 4.2%, while Johar et al. (2021) [16] found a 3.8% confirmed rate in diseased animal isolates. Islam et al. (2022) [23] reported ESBL production in 11.86% of *E. coli* and 22.2% of *K. pneumoniae* isolates, yielding an overall confirmed ESBL rate of 13.2%. Low confirmed prevalence (2.2%) was noted in a study by Eltai et al. (2018) [20]. Al-Hadidi et al. (2022) [15] described a presumptive rate of 26.7%, based on molecular screening. Gomez et al. (2021) [18] detected *bla*_TEM_ (5%) and *bla*_SHV_ (57%), while Rahman et al. (2025) [24] reported *bla*_CTX-M_, and Johar et al. (2023) [28] identified *bla*_VEB_, *bla*_GES_, and *bla*_OXA_ variants. However, without phenotypic AST, these findings remain presumptive. The meta-analysis estimated the overall prevalence of ESBL resistance to be 8% (as shown in Figure 10), with no significant heterogeneity among studies (Cochran’s Q = 3.15, *p* = 0.53; I^2^ = 0%), indicating consistent resistance levels across different study settings. The investigations on ESBL prevalence were moderately sized and showed overall moderate study quality, with some results being presumptive due to reliance on molecular detection. Sensitivity analysis for ESBL resistance showed that heterogeneity remained low, with I^2^ values ranging from 12% to 30% when individual studies were sequentially excluded. Cochran’s Q value also changed minimally, indicating stable model performance. This suggests the robustness of the results.

#### 2.5.6. Prevalence of Multidrug Resistance

**Figure 11:**
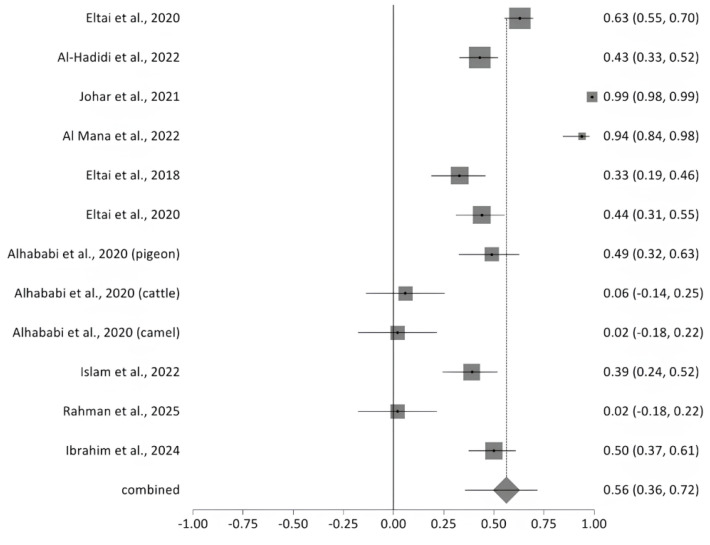
The forest plot shows MDR prevalence across studies with 95% confidence intervals. The Y-axis shows the studies investigated, and X-axis shows the correlation coefficient (r). The squares represent the resistance rate for each study, with horizontal lines depicting the 95% confidence intervals. The diamond represents the overall pooled resistance rate [14,15,16,17,20,21,22,23,24,27].

A meta-analysis of ten studies revealed substantial variability in the prevalence of multidrug resistance (MDR) among bacterial isolates, as illustrated in the forest plot (Figure 2). Reported MDR rates ranged from 2% to 99.3%. The pooled MDR prevalence, represented by the diamond at the bottom of the plot, was 56% (95% CI: 36–72%), indicating a high overall burden of resistance. Significant heterogeneity was observed across studies (Cochran’s Q = 311.147, df = 11, *p* < 0.0001), with an I^2^ value of 96.5%, suggesting that the variability is largely attributable to real differences in study populations, sample sources, or methodologies rather than chance (Figure 11). The included MDR studies were heterogeneous in sample origin and size, with moderate methodological concerns across studies. Sensitivity analysis performed for multidrug resistance across the 12 included studies showed the I^2^ values to be high and consistent (93–97%), indicating that heterogeneity was uniformly distributed and not driven by any single study. Although Cochran’s Q values showed a modest reduction when one study was removed, the direction and magnitude of the pooled effect did not change materially. This pattern suggests that while some between-study variability originated from that individual dataset, the overall results were robust, reproducible, and not overly influenced by any single study.

#### 2.5.7. Most Common Reported Isolate

**Figure 12:**
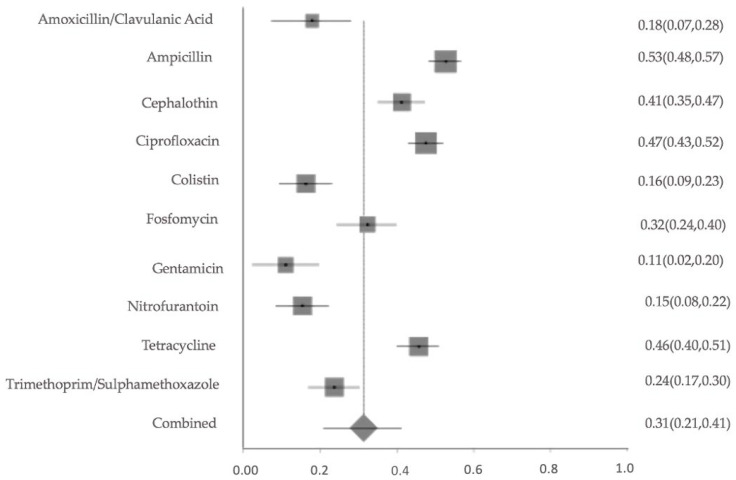
Forest plot of pooled resistance rates for *Escherichia coli* isolates across studies with 95% confidence intervals. The X-axis shows the correlation coefficient (r); the Y-axis shows the antibiotic studied. The squares represent the resistance rate for each study, with horizontal lines depicting the 95% confidence intervals. The diamond represents the overall pooled resistance rate.

*E. coli* was the most reported isolate, as reported earlier, and the resistance to ampicillin, ciprofloxacin, and tetracycline was moderately high across the included studies, with pooled prevalence estimates of 53%, 47% and 46%, respectively. The meta-analysis results for *E. coli*, the most frequently reported isolate in this systematic review, revealed significant heterogeneity (Q = 213.77, df = 9, *p* < 0.0001), with a between-study variance of 0.03186. The I^2^ statistic was 95.8% (95% CI: 94.4–96.7%), suggesting considerable inconsistency among studies in reported resistance rates. (Figure 12). The overall pooled resistance across all antibiotics was estimated at 31% (95% CI: 0.21–0.41). The studies reporting *E. coli* isolates were mostly small to moderate in scale, with moderate study quality, though resistance estimates varied substantially between studies. The sensitivity analysis of the pooled resistance for *E. coli* isolates showed very consistent I^2^ values and Cochran’s Q value. This shows that the results are robust.

## 3. Discussion

AMR poses a significant global health threat to humans, animals, and the environment [1]. Antimicrobials used in livestock and food production drive resistance, reducing treatment options and increasing health risks [30]. This is the first comprehensive meta-analysis of AMR and ARGs in non-human sources in Qatar, including animals, food, and the environment. The findings provide critical insights into the national AMR landscape beyond the clinical settings and complement a previous study focused exclusively on human-related AMR trends [13].

Despite the growing importance of One Health surveillance, only 15 eligible studies met the inclusion criteria, reflecting a significant research gap and limited surveillance coverage across non-human sectors in Qatar. Notably, the majority of these studies focused on human samples, highlighting the paucity of data from animals, food, and environmental sources and underscoring the need for expanded non-human AMR surveillance. This underlines the urgent need for integrated national AMR monitoring programs encompassing the animal, food, and environmental domains, aligned with the One Health approach. The majority of isolates analyzed were *E. coli*, consistent with previous findings reported by Ayoub et al. (2025) [31] and Akwongo et al. (2025) [32]. Region wise, high levels of antimicrobial resistance were detected in *E. coli* isolated from seawater and biota in Kuwait’s marine environment, including resistance to multiple frontline antibiotics such as third- and fourth-generation cephalosporins [33]. *E. coli* is a key One Health indicator due to its ubiquity in humans, animals, and the environment, as well as its role as a reservoir for multiple ARGs. Its presence across animal populations, food products, and environmental sources increases the risk of cross-sectoral transmission, particularly in regions with high population density, limited sanitation infrastructure, and extensive antimicrobial use [34,35]. Other priority pathogens, such as *Salmonella* spp., *Klebsiella* (*K*) *pneumoniae*, *Acinetobacter* spp., *Staphylococcus* (*S*) *aureus*, and MRSA were notably underrepresented, limiting the broader understanding of AMR threats.

WHO classifies antimicrobial agents used in human medicine as Critically Important, Highly Important, or Important, based on their therapeutic relevance and the public health risk of resistance emergence [36]. In 2017, the WHO introduced the AWaRe classification that grouped antibiotics into Access, Watch, and Reserve categories to guide optimal antibiotic use and reduce inappropriate prescribing [37]. This classification provides a stewardship tool for both human and animal health sectors to preserve antibiotic efficacy and reduce the selection pressure that drives resistance.

Our findings demonstrate high resistance levels to both Access and Watch group antibiotics, many of which are also classified as CIAs. Among the drugs in the Access group, ampicillin and tetracycline showed the highest resistance rates, approximately 50%. These results are consistent with international patterns that consider tetracyclines to be the most widely used class of antimicrobials in veterinary medicine, as stated in the World Organization for Animal Health report [38], which is often applied for growth promotion and disease prevention in livestock since the 1950s [39]. In low-resource settings, overuse of tetracyclines, penicillins, and sulfonamides in food animals remains prevalent and poorly regulated [40], exacerbating the spread of resistance. Globally, it is predicted that penicillin and tetracycline- resistant will exceed 50% in the coming years [39].

Among the Watch group antibiotics, ciprofloxacin and fosfomycin showed resistance rates of 40% and 26%, respectively. Ciprofloxacin is classified as a Highest Priority CIA due to its dual use in human and animal health and its potential to rapidly promote the development of resistant bacterial strains. Resistance to other Access or Watch antibiotics, including cephalothin (38%), trimethoprim-sulfamethoxazole (37%), amoxicillin-clavulanic acid (31%), and nitrofurantoin (28%), also restricts available treatment options. Encouragingly, relatively low resistance was noted for gentamicin (12%), ceftriaxone (9%), colistin (14%), and Carbapenems (11%). However, even low resistance levels to Reserve group antibiotics, such as colistin and carbapenems, are alarming. Colistin, classified as a Highest Priority CIA by both the WHO and the World Organization for Animal Health WOAH, is a last-resort treatment for multidrug-resistant Gram-negative infections. While colistin has been banned for veterinary use in some countries, it remains listed as essential in veterinary medicine by WOAH [38]. Continued veterinary use in the region, especially in poultry production, increases the risk of resistance development and the spread of *mcr* genes. Some studies reported alarmingly high colistin resistance among poultry isolates in specific cases. The study by Al Mana et al. (2022) [17] was excluded from the pooled colistin analysis due to the intentional pre-selection of isolates for colistin resistance, which could bias overall prevalence estimates for colistin-resistant strains. Notably, the use of colistin in poultry has been restricted in Qatar since 2021, according to personal communication with officials from the Animal Health Section of the Ministry of Municipality, Qatar. Although no official policy document confirming this date has been publicly released, the restriction is currently being implemented at the farm level. This aligns with Qatar’s National Antimicrobial Resistance Action Plan (2024–2030) for Qatar (https://cdn.who.int/media/docs/default-source/antimicrobial-resistance/amr-spc-npm/nap-library/qatar-national-antimicrobial-resistance-action-plan-2024-2030.pdf?sfvrsn=5e8e8f84_3&download=true (accessed on 7 October 2025), which outlines policies aimed at restricting the use of critically important antibiotics, such as colistin, in the animal health sector. According to studies on poultry, including the high detection rate of the *mcr-1* gene reported by Eltai et al. (2020) [14] for the first time in the Middle East, including Qatar, this restriction represents a critical step toward improving antimicrobial stewardship. However, further research is needed to assess the long-term impact of this policy on resistance trends in both animal and human populations.”.

Furthermore, a study in the UAE found ESBL-resistant *E. coli* in 23.65% of healthy pet cats and dogs, with most isolates being multidrug-resistant, including resistance to critically important antibiotics like fluoroquinolones and third- and fourth-generation cephalosporins [41], emphasizing the urgent need for strict antimicrobial stewardship programs across GCC countries to ensure appropriate long-term antibiotic use and effective monitoring.

Sector-wise, poultry emerged as the most significant AMR reservoir, with the highest prevalence of MDR and resistance to multiple Watch group, and critically important antibiotics. For example, several poultry isolates demonstrated complete resistance to ciprofloxacin. In contrast, other animals, such as cattle, sheep, and camels, generally showed lower levels of AMR, which may suggest less intensive antimicrobial use; however, this cannot be confirmed due to the limited number of studies conducted on these animal species. Interestingly, AMR was also detected in semi-wild species like the Arabian oryx, highlighting environmental spillover that may be due to human activity or contaminated feed and water sources. In Kuwait, *Salmonella* from poultry farms and processing plant environments showed a prevalence of 4.7–5.4%, with *Salmonella Enteritidis* being most common, and all isolates exhibiting resistance to at least one antibiotic, particularly ampicillin, nalidixic acid, and tetracycline, highlighting the need for prudent antibiotic use [42]. In Saudi Arabia, MRSA has been detected in both food-producing animals and retail food products, with livestock-associated strains showing virulence traits that increase the risk of human infection [43]. Another pilot study in Saudi Arabia found that MRSA and methicillin-resistant *Staphylococcus epidermidis,* from goats and their farm environments, carry genotypes and plasmids previously linked to human infections, harbor key virulence genes, and show resistance to trimethoprim-sulfamethoxazole, indicating potential cross-transmission between animals, the environment, and humans [44]. Moreover, a study in the UAE on pets, ESBL-producing, multidrug-resistant *E. coli* were found to carry the colistin resistance gene mcr-1.1, with genomic links to chicken meat isolates, highlighting pets as potential reservoirs of AMR and the need for a One Health surveillance approach [45]. Failure to address the root causes of animal diseases can promote AMR, highlighting the need for judicious antibiotic use in animal production and treatment [46].

In food samples, resistance to ampicillin, tetracycline, and trimethoprim-sulfamethoxazole (Primarily Access drugs) was particularly evident in poultry meat and seafood products. However, the lack of clarity regarding the origin (imported vs. locally sourced) of many food items limited a conclusive interpretation. Given Qatar’s heavy reliance on food imports, robust monitoring of imported animal products is essential to prevent the transboundary introduction of resistant pathogens and ARGs. In Saudi Arabia, *E. coli* isolated from raw milk exhibited lower antibiotic resistance and no ESBL production, indicating that raw milk can be a potential, though less significant, source of resistant *E. coli* in the food chain [47]. Moreover, a review study in the Middle East showed that foodborne pathogens, including *E. coli*, *Salmonella* spp., *S. aureus*, and *Listeria* spp., exhibit increasing antimicrobial resistance, particularly to ampicillin, amoxicillin-clavulanic acid, nalidixic acid, streptomycin, and tetracycline, while surveillance and standardization of testing remain limited across the region [48]. A study about the retail foods in the UAE, especially chicken and camel meat, was found to harbor multidrug-resistant MRSA carrying *mecA* and various toxin genes (mainly **sea** and exfoliative toxin A), highlighting the role of the food environment as a reservoir for AMR and virulent strains [49]. Interestingly, in efforts to remove antibiotics from food products, a Saudi study demonstrated that ozonation efficiently degraded antibiotics in milk, producing harmless fragments and eliminating their antimicrobial activity [50]. Subsequently, microbial food safety and AMR pose a critical global health threat, requiring coordinated One Health strategies and robust surveillance [51]. It is significant to note that maintaining proper farm hygiene is essential to prevent the spread of pathogens, reduce bacterial contamination in food products, and minimize the emergence and transmission of antimicrobial-resistant bacteria in food-animal environments [52]. On the other hand, a study on fresh vegetables in the UAE showed contamination with *E. coli*, with a notable proportion of isolates exhibiting multidrug resistance and carrying genes such as blaCTX-M-15 and virulence factors, highlighting the potential risk of foodborne illness and AMR transmission through fresh produce [53]. To our knowledge, no similar studies on fresh vegetables have been conducted in Qatar.

The environmental sector, including samples from water, air, and public toilets, also revealed concerning resistance rates, particularly to ampicillin and metronidazole. Opportunistic Gram-negative pathogens such as *P. aeruginosa* and *Acinetobacter* spp. are known for intrinsic resistance and environmental resilience, and were frequently identified [23,26,27,28]. These organisms pose particular risks in healthcare-adjacent settings and wastewater streams [54,55]. Hospital effluents and municipal wastewater were implicated as major sources of AMR dissemination [27,28]. This aligns with global findings showing significant AMR gene load in treated wastewater entering the environment [56]. As well as the conclusions from this KSA study emphasize the critical role of wastewater in sustaining and disseminating AMR [57]. In addition, a study performed in a hospital and municipal sewage in southwestern Saudi Arabia harbors KPC, vanA/B, and mecA genes, highlighting the environment as a reservoir for antibiotic-resistant bacteria [58]. It is worth noting that two studies had a very high resistance to metronidazole [25,26], raising concerns due to the antibiotic’s common use in treating infections, which can lead to treatment failure, such as bacterial vaginosis [59]. This high resistance suggests environmental bacteria may act as reservoirs for resistance genes, potentially compromising treatment effectiveness. Moreover, a rapid baseline survey of 560 *E. coli* from seawater across four GCC states revealed 32.5% multi-drug resistance, high reduced susceptibility to key antibiotics, and frequent carriage of *qnrS1* and *blaCTX-M-15*, highlighting environmental AMR and the need for regional marine surveillance [60]. Furthermore, airborne AMR bacteria can be detected, as demonstrated by the study in Riyadh restaurants, where aerosols from food preparation, packaging, and handwashing zones harbored *E. coli*, *S. aureus*, and aerobic bacteria with varying levels of antibiotic resistance, highlighting the role of air as a reservoir and transmission route for AMR in food environments [61]. Similarly, in Kuwait, indoor and outdoor aerosols—including hospital environments- were found to contain a diverse array of ARGs, including beta-lactam, aminoglycoside, fluoroquinolone, tetracycline, MLSB, multidrug-resistant, and vancomycin-resistant genes, confirming aerosols as a significant vehicle for ARG dissemination among human and non-human biota [62]. To the best of our knowledge, only two studies have investigated airborne AMR in Qatar, indicating that further research is still required.

At the genetic level, several ARGs were detected across sample types, including *bla*_CTX_*-*_M_, *bla*_TEM_, and *bla*_SHV_, which encode ESBLs. Other important ARGs included *mcr-1* (colistin resistance), *qnr* (quinolone resistance), and macrolide resistance genes such as *ermB* and *mefA*. The detection of these genes in non-clinical settings indicates potential for horizontal gene transfer and MDR development, especially in microbial communities exposed to selective pressures from subtherapeutic antibiotic concentrations [63,64]. In Saudi aquaculture, widespread ARGs, mainly resistant to beta-lactams, penicillin, quinolones, and tetracyclines, are driven by antibiotic use, poor sanitation, biofilms, and contaminated feed, emphasizing the need for better management, research, and public awareness [65]. Another Saudi study investigated clinical and environmental samples discovered multi-drug resistant *Salmonella enterica* carrying key resistance genes, including carb-like, *dfrA1*, *floR*, *tetA*, *gyrA*, and *parC*, highlighting environmental reservoirs of antimicrobial resistance [66].

In Kuwait, AMR is widespread across humans, animals, and the environment, with poultry showing high resistance to cefotaxime, ampicillin, and amoxicillin, camel milk isolates resistant to penicillin, tetracyclines, and carbapenems, and environmental samples harboring diverse ARGs such as ESBLs, carbapenemases, and colistin resistance; the overlapping of these resistance genes across domains and high meta-analysis resistance rates (humans 34.05%, animals 67.42%, environment 69.86%) highlight the interconnected nature of AMR, emphasizing the urgent need for integrated One Health interventions in Kuwait [67].

MDR was common across isolates, with a prevalence of 64%, particularly in poultry, where rates exceeded 90% in several studies, such as Johar et al. (2021) study [16]. However, inconsistent reporting methods and definitions of MDR across studies limit direct comparison and underscore the need for standardized surveillance protocols. Across the Arabian Peninsula, a review of 382 human and animal health studies across nine Arabian Peninsula countries showed over 120 emerging MDR microbes, with *A. baumannii*, *M. tuberculosis*, and *S. aureus* causing most MDR-related deaths and a 16.5% overall mortality rate [68]. In Saudi Arabia, peregrine falcons were found to carry multidrug-resistant *Pseudomonas aeruginosa* harboring β-lactamase, aminoglycoside, fosfomycin, and chloramphenicol resistance genes, with genomic similarity to human strains, highlighting their zoonotic potential and the need for One Health–based AMR surveillance [69]. However, the evidence included in this review has notable limitations. Most studies were observational with small and heterogeneous sample sizes, which may affect the precision and generalizability of AMR estimates. Several studies exhibited insufficient control for confounding factors and variability in sampling methods, laboratory protocols, and antibiotic susceptibility testing (AST) standards. These inconsistencies contributed to high heterogeneity, particularly among food and environmental samples. The high level of heterogeneity (I^2^ often >90%) likely reflects multiple underlying factors beyond sampling variation. These include differences in AST methods (e.g., disk diffusion vs. broth microdilution), interpretive standards (CLSI vs. EUCAST), and resistance breakpoints applied across studies. Temporal variability in data collection periods, differences in species sampled, ecological contexts, and antimicrobial exposure histories also contribute substantially to the observed heterogeneity. Moreover, variations in environmental matrices (soil, water, food, or air), biosecurity measures, and regional management practices may influence microbial load and resistance profiles. This diversity is inherent to One Health AMR research and underscores the need for standardized methodologies and metadata reporting to improve comparability in future analyses. Furthermore, incomplete reporting of study settings, isolates, and resistance outcomes reduced comparability and reproducibility. These limitations highlight the urgent need for harmonized surveillance frameworks by means of standardized methodologies and detailed metadata reporting.

This review process itself also has limitations. Despite a comprehensive search strategy across multiple databases and grey literature sources, unpublished data and studies in non-English languages may have been missed. The small number of eligible studies limited the ability to perform subgroup analyses or meta-regressions to explore heterogeneity. Additionally, due to data scarcity, we relied on descriptive synthesis for some sectors rather than quantitative pooling, which may restrict broader generalization.

The implications of these findings for policy and practice are substantial. Strengthening Qatar’s AMR surveillance system under a One Health framework is essential to address current data gaps and support evidence-based decision-making. Integration of molecular AMR and ARG monitoring into routine veterinary and environmental programs should be prioritized. Policies restricting the use of critically important antibiotics, particularly those in the Watch and Reserve groups, should be rigorously enforced, coupled with awareness campaigns for antimicrobial stewardship among veterinarians and farmers. Future research should focus on longitudinal studies that assess the effectiveness of these interventions, monitor trends in ARG dissemination, and explore genomic linkages between human and non-human isolates to inform cross-sectoral control strategies. Moreover, it further emphasizes the need to conduct a future comparative study between Qatar and other GCC countries to better understand and address existing gaps in regional AMR patterns.

## 4. Methods

### 4.1. Study Protocol

This systematic review followed the Preferred Reporting Items for Systematic Reviews and Meta-Analyses (PRISMA) guidelines [70] to ensure a structured and transparent approach to study identification, selection, and analysis. The PRISMA framework proposed a detailed checklist and flow diagram to document the inclusion and exclusion process, reducing bias and improving reproducibility.

### 4.2. Search Strategy

A comprehensive literature search was conducted across multiple databases, including PubMed, Scopus, Web of Science, Embase, and Google Scholar (limited to the first three pages to capture the most relevant in gray literature and local reports not indexed in major databases, to identify relevant studies on AMR in animals, food, and the environment in Qatar. The search was performed on 18–19 February 2025, with no restrictions on publication dates to ensure comprehensive coverage of all relevant literature. The search was tailored to each database using Boolean operators (AND, OR), and the following search terms were used: “antimicrobial resistance, AMR, antibiotic resistance, animals, livestock, poultry, cattle, food, dairy, meat, environment, wastewater, sea, soil, water, air, Qatar, Doha. The following search terms were executed via the Boolean operator NOT: “Saudi Arabia, UAE, Kuwait, Bahrain, Oman, Iran, Middle East”. Furthermore, a search of the Qatar Research, Development, and Innovation (QRDI) database on the Qatar National Research Fund (QNRF) website was conducted to identify any potentially relevant studies that may have been funded, published as preprints, or not yet released. This confirmed that all relevant studies had already been captured through the primary database search. Moreover, an article published in March 2025, after the search, was identified and manually added. Finally, the reference lists of the included studies were also manually screened for any additional eligible articles. Detailed database search strategies are provided in Supplementary Section S1.

### 4.3. Screening and Eligibility of Studies

Study selection involved a two-step screening: initial title and abstract review, followed by full-text assessment based on predefined criteria.

#### Inclusion Criteria and Exclusion Criteria

Inclusion Criteria: research articles and dissertations reporting AMR/ARG in animals, food, or the environment in Qatar that encompassed phenotypic and/or genotypic AMR data were included. Exclusion Criteria: All studies focusing on human clinical samples or human AMR surveillance were excluded from this systematic review. Additionally, studies conducted outside of Qatar, as well as commentaries and studies that examine antibiotic usage without associated resistance data. Non-primary research articles, including book chapters, review articles, conference proceedings, and poster presentations, were excluded.

### 4.4. Data Extraction

Following a comprehensive search of electronic databases, the retrieved studies were imported into Zotero (version 6.0.36) for citation management and duplicate removal. Any remaining duplicate entries caused by inconsistencies in citation formats between databases and indexing systems were manually corrected. Reviewer (LA) independently conducted the identification and screening process of titles and abstracts, per the inclusion/exclusion criteria. Second reviewer (RK) verified the included and excluded studies. Any discrepancies were resolved through discussion with a third reviewer (NE). Reviewer (LA) autonomously retrieved the full texts of the selected papers and assessed them for eligibility and quality. Data were extracted and collected using a standardized Microsoft Excel 365 data abstraction form including: author(s), year of publication, journal/source, geographic location, study design, sample size, sampling period, sample type (animals, food, or environment), bacterial isolates, antimicrobials tested, AMR testing methods (phenotypic or genotypic method, resistance prevalence (%), multidrug resistance (MDR) detection, resistance mechanisms (e.g., Extended-Spectrum Beta-Lactamases (ESBL), carbapenems and finally, the key findings and public health relevance. Additional variables included study design and detection methods when available. When data were unclear, calculations were made based on contextual information provided in the article or corresponding data from similar studies. The second reviewer (RK) rechecked the extracted data in the full-text assessment and confirmed the accuracy of the involved and extracted data.

### 4.5. Statistical Analysis and Meta-Analysis

Statistical and meta-analyses were conducted to assess AMR patterns across studies. GraphPad Prism (v10.4) was used to calculate the average resistance percentage for each antimicrobial agent, followed by pooling resistance percentages across all included studies (Table S1). Results were stratified by source type: animals, food, and the environment. Unless otherwise stated, unweighted averages were used, treating all studies equally without adjustment for sample size; studies without quantitative resistance data were excluded. Gram-positive and Gram-negative isolates were pooled in the analysis, as only one study reported Gram-positive data, and the antimicrobial panels included shared agents such as ampicillin and ciprofloxacin; therefore, this dataset was not differentiated or excluded.

A descriptive narrative was performed for data where meta-analysis was not possible. In addition, a series of meta-analyses was done to formally estimate pooled resistance outcomes, including the most commonly studied antibiotics, AMR prevalence stratified by source type (animals, food, environment), and resistance to clinically important and less critical antibiotics such as carbapenems, colistin, ciprofloxacin, fosfomycin, and ampicillin. Colistin resistance data from one study [17] were excluded from quantitative analysis, as all isolates were pre-selected for colistin resistance, which could bias the overall prevalence estimate. Meta-analyses were also made for the prevalence of ESBL production and multidrug resistance (MDR).

Meta-analyses were conducted using StatsDirect Statistical Analysis Software, version 4.0.4 (Altrincham, UK), applying weighted random-effects models depending on heterogeneity, which was assessed using Cochran’s Q and the I^2^ index. Forest plots were generated for each outcome. Publication bias has been evaluated for outcomes with 10 or more studies using Egger’s regression test for funnel plot asymmetry. All analyses were stratified by antibiotic class or resistance mechanism to enhance comparability.

Study quality and risk of bias were assessed by one reviewer using the Joanna Briggs Institute (JBI) Critical Appraisal Checklist for Prevalence Studies. No automation tools were used. The certainty of evidence was assessed based on study quality, consistency, and heterogeneity, acknowledging limitations from small study numbers and methodological variability. Sensitivity analysis was performed using the leave-one-out approach, in which the meta-analysis was repeated by sequentially removing one study at a time to evaluate the influence of individual studies (StatsDirect Statistical Analysis Software, version 4.0.4, Altrincham, UK). Changes in the pooled estimates, heterogeneity (I^2^), and Cochran’s Q values were examined to assess the robustness of the results.

## 5. Conclusions

This review underscores the fragmented and inadequate state of AMR surveillance in Qatar’s animal, food, and environmental sectors. High resistance to commonly used Access and Watch antibiotics, along with the increasing detection of critical ARGs, points to significant gaps in current monitoring efforts. Environmental and foodborne pathways, including the imported food, livestock, and wild birds, further amplify the risk of AMR spread. To address this, a coordinated national One Health strategy is urgently required that includes harmonized AMR surveillance, routine ARG monitoring, and strict antimicrobial stewardship. Applying the WHO AWaRe framework and prioritizing CIA will be essential for guiding targeted interventions and protecting public health.

## Figures and Tables

**Table 1:** Characteristics of selected studies on antibiotic resistance in non-human samples from different databases from the time of inception to February 2025.

Study	Author	Studied Sample Category/Type	Study Design	Sampling Period	Sample Size	Studied Bacteria	AMR Phenotypic Assay	Key Finding	Public Health Risk
S1: Prevalence of antibiotic-resistant *Escherichia coli* isolates from local and imported retail chicken carcasses	Eltai et al., 2020 [14]	Food products (animal-derived)/chicken carcass rinse	Cross-sectional study	Nov. 2017–Apr. 2018	270	*E. coli*	Disc diffusion and double disk synergy test	MDR, ESBL, and colistin-resistant isolates were significantly more prevalent in local chilled chicken than in imported chilled and frozen samples.	The co-existence of MDR and colistin-resistant *E. coli* in chicken meat may reduce treatment efficacy and increase the risk of AMR transmission through the food chain.
S2: Retail Chicken Carcasses as a Reservoir of Multidrug-Resistant *Salmonella*	Al-Hadidi et al., 2022 [15]	Food products (animal-derived)/chicken carcass rinse	Cross-sectional study	Nov. 2017–Apr. 2018	270	*Salmonella*	Disk diffusion and E-test	MDR *Salmonella* was more common in frozen chicken; ceftriaxone and amoxicillin-clavulanic acid resistance were higher in frozen imported samples.	MDR *Salmonella* in poultry highlights a tangible threat of foodborne transmission of resistance to consumers, potentially leading to infections that are difficult to treat.
S3: Antibiotic Resistance and Virulence Gene Patterns Associated with Avian Pathogenic *Escherichia coli* (APEC) from Broiler Chickens in Qatar	Johar et al., 2021 [16]	Food products (animal-derived)/chicken carcass (air sacs, cloacal, kidney, liver, and trachea)	Cross-sectional study	Sep. 2020–Dec. 2020	47	*E. coli*	Disk Diffusion and E-test	High prevalence of antibiotic-resistant *E. coli* in both healthy and diseased chicken carcasses reflects persistent AMR in poultry.	Widespread resistant *E. coli* in poultry reflects a stable AMR source that may spread to humans via the food production and handling pipeline.
S4: Transmissibility and Persistence of the Plasmid-Borne Mobile Colistin Resistance Gene, mcr-1, Harbored in Poultry-Associated *E. coli*	Al Mana et al., 2022 [17]	Food products (animal-derived)/chicken carcass rinse	Cross-sectional study	Sep. 2016–Jan. 2017 and Nov. 2017 to Apr. 2018	18	*E. coli*	E-test method.	The *mcr-1* gene was plasmid-borne, transmissible via conjugation, found in diverse genetic backgrounds, and reduced colistin resistance in biofilms over time.	Plasmid-mediated *mcr-1* gene in *E. coli* poses a risk of horizontal transmission of colistin resistance, compromising critical last-resort therapies.
S5: Prevalence of antibiotic-resistant pathogenic *E. coli* from animals, retail, and humans diagnosed with Gastroenteritis.	Gomez et al., 2021 [18]	Food products (animal-derived)/beef, mutton, chicken, and seafood	Cross-sectional study	NR	Retail isolates 56	*E. coli*	Disk diffusion test	*E. coli* from animals, meat processing plants, retail, and humans revealed AMR through phenotypic and genotypic testing.	The detection of MDR *E. coli* throughout food processing environments signifies environmental persistence, posing risks for contamination and public exposure.
S6: Multiple Antibiotic Resistance (MAR), Plasmid Profiles, and DNA Polymorphisms among *Vibrio vulnificus* Isolates	Al-Dulaimi et al., 2019 [19]	Food products (animal- derived)/seafood (clams)	Cross-sectional study	Jul. 2013–Feb. 2014	Qatar samples 23	*Vibrio vulnificus*	Disc diffusion test	*V. vulnificus* isolates exhibited a high MAR index, suggesting strong environmental antibiotic pressure in seafood sources.	High MAR indices and genomic variability in *V. vulnificus* suggest that seafood consumers are at elevated risk of severe infection and limited treatment options.
S6: Antibiotic Resistance Profile of Commensal *Escherichia coli* Isolated from Broiler Chickens in Qatar	Eltai et al., 2018 [20]	Animals/chicken cloacal swabs	Cross-sectional study	Sep. 2016–Jan. 2017	172	*E. coli*	E-test and double-disc synergy test.	High prevalence of antibiotic-resistant *E. coli* in food-producing animals may reflect prolonged antibiotic use in agriculture in Qatar.	The transfer of resistant bacteria through unhygienic food handling practices can escalate AMR spread and increase risks for vulnerable populations.
S7: Antibiotic resistance profile of commensal *Escherichia coli* isolated from healthy sheep in Qatar	Eltai et al., 2020 [21]	Animals/sheep rectal swabs	Cross-sectional study	Dec. 2016–Jul. 2017	171	*E. coli*	E-test method	MDR *E. coli* was found in rectal swabs of sheep, indicating possible zoonotic transmission and environmental persistence.	Zoonotic pathogens resistant to multiple drugs can be transmitted through food or contact, posing broader public health threats beyond localized outbreaks.
S8: Antimicrobial Resistance of Commensal *Escherichia coli* Isolated from Food Animals in Qatar.	Alhababi et al., 2020 [22]	Animals/camels, cattle, and pigeons fecal samples	Cross-sectional study	Dec. 2018–Feb. 2019	300	*E. coli*	Disk diffusion and E-test method	70.7% of pigeon isolates showed resistance; 50% were MDR, raising concerns about their role in spreading AMR in urban environments.	High MDR levels in urban pigeons indicate a potential AMR reservoir, increasing the risk of interspecies transmission in densely populated areas.
S9: Diversity of bacterial pathogens and their antimicrobial resistance profile among commensal rodents in Qatar.	Islam et al., 2022 [23]	Animals/rodents’ intestinal contents	Cross-sectional study	Aug. 2019–Feb. 2020	148	Mixed culture	VITEK^®^ 2 AST-GN and VITEK^®^ 2 AST-GN 85	MDR pathogens were not linked to rodent species or location; 11.86% of *E. coli* and 22.2% of *K. pneumoniae* were ESBL-producers.	Rodents carrying MDR and ESBL-producing bacteria can serve as stealthy AMR vectors, affecting human and animal health through environmental or direct contact.
S10: A Snapshot of Antimicrobial Resistance in Semi-Wild Oryx: Baseline Data from Qatar	Rahman et al., 2025 [24]	Animals/semi-wild Arabian Oryx (Oryx leucoryx) rectal faecal	Cross-sectional study	Feb. 2024–May. 2024	100	*E. coli*	Disk diffusion test	Low AMR in semi-wild oryx; resistance was mainly to tetracycline, with some isolates harboring ESBL and efflux pump genes.	MDR organisms in oryx imply possible environmental AMR exposure, highlighting the interconnectedness of wildlife and human health ecosystems.
S11: Seasonal and spatial variations in concentration, diversity, and antibiotic resistance of ambient bioaerosols in an arid region	Sajjad et al., 2023 [25]	Environment/ambient air bioaerosols	Cross-sectional study	Nov. 2021–Oct. 2022	300	Mixed culture	Disk diffusion test	All bacterial isolates in the study showed 100% resistance to metronidazole, with AMR levels peaking in the humid-hot summer.	Even one MDR isolate with MAR > 0.2 signals contact with high-risk AMR zones, alerting to potential environmental or animal–human AMR pathways.
S12: Size-resolved ambient bioaerosols concentration, antibiotic resistance, and community composition during autumn and winter seasons in Qatar	Sajjad et al., 2024 [26]	Environment/ambient air bioaerosols	Cross-sectional study	Oct. 2021–Jan. 2022	156	Mixed culture	Disk diffusion test	Frequent resistance was observed to metronidazole, ampicillin, ciprofloxacin, and trimethoprim-sulfamethoxazole across environmental samples.	Metronidazole-resistant airborne bacteria challenge current disinfection protocols and indicate environmental AMR spread, especially during warmer seasons.
S13: Surveillance of Bacterial Load and Multidrug-Resistant Bacteria on Surfaces of Public Restrooms	Ibrahim et al., 2024 [27]	Environment/public restrooms surfaces (seat, water sprayer, tap, inner door handle, outer door handle)	Cross-sectional study	NR	160	Mixed culture	BD Phoenix™ automated microbiology system	MDR *Staphylococcus haemolyticus* and *Staphylococcus kloosii* were detected in multiple samples, highlighting emerging resistance in non-enteric bacteria.	Resistance to common antimicrobials like ciprofloxacin and SXT in environmental samples undermines frontline treatment strategies for infections.
S14: Wastewater-based epidemiology for tracking bacterial diversity and antibiotic resistance in COVID-19 isolation hospitals in Qatar	Johar et al., 2023 [28]	Environment/waste-water	Cross-sectional study	May 2021–Nov. 2021	12	Mixed culture	NA	Hospital wastewater contained 61 of 87 tested ARGs (88.5% at the COVID-19 site), with dominant ARGs including β-lactamase and fluoroquinolone resistance genes.	The presence of ARGs in hospital wastewater raises the alarm for AMR dissemination into public water supplies, endangering both human and ecological health.

NR: not reported, NA: not applicable.

## Data Availability

All data are included in this study either in the manuscript or in the Supplementary Section.

## Supplementary Materials

The following supporting information can be downloaded at: https://www.mdpi.com/article/10.3390/antibiotics14121219/s1, Figure S1. AMR rates (%) of bacterial isolates isolated from animals. Figure S2. AMR rates (%) of bacterial isolates isolated from food products. Figure S3. Antimicrobial resistance rates (%) of bacterial isolates isolated from environmental samples. Table S1. Pooled average resistance percentage across included studies. Table S2. Studied bacterial isolates in all 15 studies. Table S3. Used the Abs panel in all 15 studies. Table S4. Detected ARGs in the included studies.

## Abbreviations

The following abbreviations are used in this manuscript:

AMR	Antimicrobial Resistance
Abs	Antibiotics
ARGs	Antimicrobial Resistance Genes
AST	Antimicrobial Susceptibility Testing
AWaRe	Access, Watch, and Reserve (WHO classification of antibiotics)
CIA	Critically Important Antimicrobials
ESBL	Extended-Spectrum Beta-Lactamase
GCC	Gulf Cooperation Council
GCC-IC	Gulf Cooperation Council Center for Infection Control
GLASS	Surveillance System
MDR	Multidrug Resistance/Multidrug-Resistant
MRSA	Methicillin-Resistant *Staphylococcus aureus*.
N/A	Not Applicable
N/R	Not Reported
One Health	Integrated approach linking human, animal, and environmental health
PCR	Polymerase Chain Reaction
TrACSS	Tracking AMR Country Self-Assessment Survey
WHO	World Health Organization
WOAH	World Organization for Animal Health (formerly OIE)
