# Supplementary material for: One Health Antimicrobial Resistance in Qatar: A Comprehensive Systematic Review and Meta-Analysis of Animal, Food, and Environmental Reservoirs
Source: Antibiotics (Basel). 2025 Dec 3;14(12):1219. doi: 10.3390/antibiotics14121219 (PMC12729573; doi:10.3390/antibiotics14121219)
Supplement: Supplementary file 1 [file antibiotics-14-01219-s001.zip › antibiotics-3976063-supplementary.pdf]

Appendix A (Supplementary Figures and Tables)

Supplementary Data:

S1. Database-specific search strategies

- PubMed:
("antimicrobial resistance" OR "AMR" OR "antibiotic resistance") AND (animals OR livestock OR poultry OR cattle OR food OR dairy OR meat OR environment OR wastewater OR sea OR air OR soil OR water) AND ("Qatar" OR "Doha") NOT (Saudi Arabia OR UAE OR Kuwait OR Bahrain OR Oman OR Iran OR "Middle East")
Results: 44 studies
- Scopus: Same as PubMed, adapted for Scopus syntax.
Results: 19 studies
- Web of Science:
TS=("antimicrobial resistance" OR "AMR" OR "antibiotic resistance") AND TS=(animals OR livestock OR poultry OR cattle OR food OR dairy OR meat OR environment OR wastewater OR sea OR air OR soil OR water) AND TS=("Qatar" OR "Doha") NOT TS=(Saudi Arabia OR UAE OR Kuwait OR Bahrain OR Oman OR Iran OR "Middle East")
Results: 17 studies
- Embase:
('antimicrobial resistance'/exp OR 'AMR' OR 'antibiotic resistance') AND ('animal'/exp OR 'livestock'/exp OR 'poultry'/exp OR 'cattle'/exp OR 'food'/exp OR 'dairy product'/exp OR 'meat'/exp OR 'environment'/exp OR 'wastewater'/exp OR 'sea'/exp OR 'soil'/exp OR 'water'/exp OR 'air'/exp) AND ('Qatar'/exp OR 'Doha') NOT ('Saudi Arabia'/exp OR 'United Arab Emirates'/exp OR 'Kuwait'/exp OR 'Bahrain'/exp OR 'Oman'/exp OR 'Iran'/exp OR 'Middle East'/exp)
Results: 368 studies
- Google Scholar:
("antimicrobial resistance" OR AMR OR "antibiotic resistance") AND (animals OR livestock OR poultry OR cattle OR food OR dairy OR meat OR environment OR wastewater OR sea OR soil OR water OR air) AND ("Qatar") NOT "Saudi Arabia" NOT "UAE" NOT "Kuwait" NOT "Bahrain" NOT "Oman" NOT "Iran" NOT "Middle East"
Results: 5,790 (first three pages included, n = 30 studies)

S2. Figures

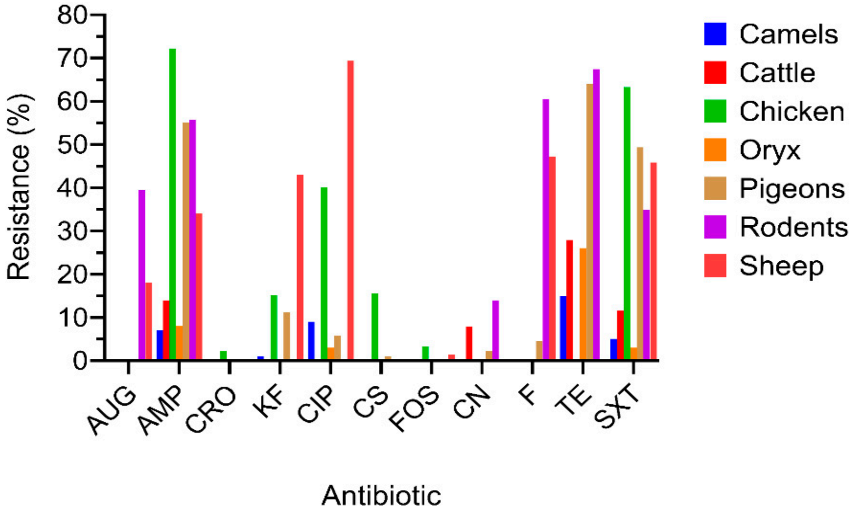

Figure S.1. AMR rates (%) of bacterial isolates isolated from animals. AUG: Amoxicillin-Clavulanic Acid; AMP: Ampicillin; CRO: Ceftriaxone; KF: Cephalothin; CIP: Ciprofloxacin; CS: Colistin; FOS: Fosfomycin; CN: Gentamicin; F: Nitrofurantoin; TE: Tetracycline; SXT: Trimethoprim-Sulfamethoxazole.

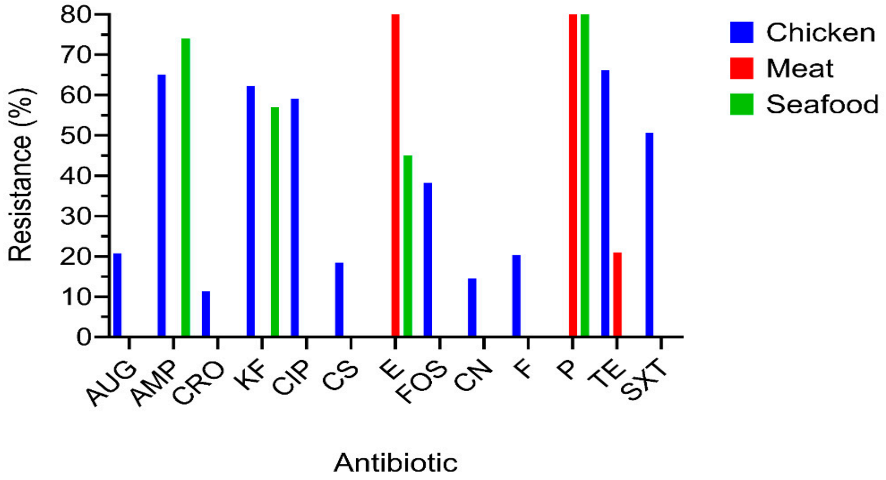

Figure S.2. AMR rates (%) of bacterial isolates isolated from food -products. AUG: Amoxicillin-Clavulanic Acid; AMP: Ampicillin; CRO: Ceftriaxone; KF: Cephalothin; CIP: Ciprofloxacin; CS: Colistin; FOS: Fosfomycin; CN: Gentamicin; F: Nitrofurantoin; P: Penicillin; TE: Tetracycline; SXT: Trimethoprim-Sulfamethoxazole.

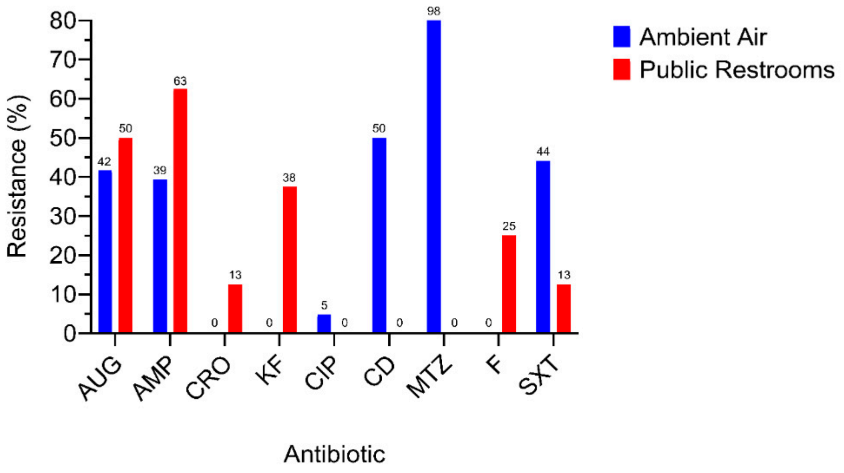

Figure S.3. Antimicrobial resistance rates (%) of bacterial isolates isolated from environmental samples. AUG: Amoxicillin-Clavulanic Acid; AMP: Ampicillin; CRO: Ceftriaxone; KF: Cephalothin; CIP: Ciprofloxacin; CD: Clindamycin; MTZ: Metronidazole; F: Nitrofurantoin; SXT: Trimethoprim-Sulfamethoxazole.

S3. Tables

Table S. 1 Pooled average resistance percentage across included studies.

Antibiotics	S 1	S 2	S 3	S 4	S 5	S 6	S 7	S 8	S 9	S 10	S 11	S 12	S 13	S 14	Average
Amikacin	0.9							0.7		7					2.8667
Amoxicillin												38.1	33.3		35.7
Amoxicillin-Clavulanic Acid	6	26.7	29.55					18		39.5		33.3	50	50	31.631
Ampicillin	52.3	50	91.2	66.7		74	72.22	34	25.4	55.8	8	28.6	50	52.6	50.832
Aztreonam														12.5	12.5
Bacitracin						52									52
Cefepime	2.3						1.11								1.705
Cefotaxime											1			25	13
Cefovecin										4.7					4.7
Cefoxitin														25	25
Cefpodoxime										41.9					41.9
Ceftiofur										34.9					34.9
Ceftriaxone	5.1	26.7	2.2				2.22							12.5	9.744
Cefuroxime	6.9		2.2				4.44							25	9.635
Cephalothin	45.4		97.05	44.4		57	15.156	43	6.1	23.3				37.5	38.9
Chloramphenicol		3.3							8.2	18.6		38.1			17.05
Ciprofloxacin	47.7	23.3	98.9	66.7			40	69.4	7.4		3	4.8			40.133
Clindamycin						61						50	50		53.667
Colistin	31.9	6.7	16.65				15.56		1						14.362
Daptomycin															
Doxycycline										23.3		2.4			12.85

Enrofloxacin										34.9					34.9
Ertapenem	0.9													25	12.95
Erythromycin					91	45								25	53.667
Fosfomycin	18.1	10	80.45	44.4			3.33	1.4							26.28
Gentamicin	15.7		11.1	16.7					5.1	14					12.52
Imipenem										27.9				25	26.45
Kanamycin						13									13
Levofloxacin												2.4			2.4
Linezolid															
Meropenem	1.4														1.4
Metronidazole												95.2	100		97.6
Moxifloxacin															
Mupirocin-High Level															
Neomycin					8										8
Nitrofurantoin	2.3	53.3		5.6				47.2	4.5	60.5				25	28.343
Novobiocin						35									35
Oxacillin														25	25
Penicillin					88	91								25	68
Piperacillin										14					14
Piperacillin/Tazobactam	4.2		0.75												2.475
Rifampin															
Streptomycin					25										25
Sulfamethoxazole	62														62
Teicoplanin															
Tetracycline	59.7	73.7	81.35	50	21				26.4	67.4	26				50.694
Tigecycline								0.7						12.5	6.6
Tobramycin										7					7
Trimethoprim	52.3														52.3
Trimethoprim-Sulfamethoxazole		6.7		94.4			63.33	45.8	22	34.9	3	38.1	50	12.5	37.073
Vancomycin						65									65

Table S.2 Studied bacterial isolates in all 15 studies.

Study	*Microorganisms Studied*
**Eltai et al., 2020**	*E. coli*
**Al-Hadidi et al., 2022**	*Salmonella*
**Johar et al., 2021**	*E. coli*
**Al Mana et al., 2022**	*E. coli*
**Gomez et al., 2021**	*E. coli*
**Al-Dulaimi et al., 2019**	*Vibrio vulnificus*
**Eltai et al., 2018**	*E. coli*
**Eltai et al., 2020a**	*E. coli*

**Alhababi et al., 2020**	*E. coli*
**Islam et al., 2022**	*Acinetobacter baumannii, Aeromonas salmonicida, Citrobacter freundii, Citrobacter koseri, Enterobacter aerogenes, Enterobacter cloacae, Escherichia coli, Hafnia alvei, Klebsiella pneumoniae, Providencia stuartii, Proteus mirabilis, Pseudomonas aeruginosa, and Salmonella enterica.*
**Rahman et al., 2025**	*E. coli*
**Sajjad et al., 2023**	*Chryseobacterium, Pseudomonas, Pantoea, Proteus, Myroides, Yersinia, Pasteurella, Ochrobactrum, Vibrio*
**Sajjad et al., 2024**	*Pasteurella pneumotropica / Mannheimia haemolytica, Pantoea spp., Proteus penneri, Myroides spp. / Chryseobacterium indologenes, Yersinia pseudotuberculosis, Yersinia enterocolitica, Ochrobactrum anthropi, Pseudomonas aeruginosa, Pasteurella aerogenes, Proteus mirabilis, Stenotrophomonas maltophilia, Chromobacterium violaceum, Providencia rettgeri, Providencia stuartii, Serratia plymuthica, Serratia rubidaea, Serratia marcescens, Pseudomonas luteola, Burkholderia cepacia, Photobacterium damselae, Aeromonas salmonicida ssp. Salmonicida*
**Ibrahim et al., 2024**	*Staphylococcus haemolyticus, Staphylococcus kloosii, Micrococcus leuteus, Staphylococcus pasteuri, Bacillus clausii, Streptomyces violaceoruber, Klebsiella pneumoniae, Pseudomonas aeruginosa, Pantoea agglomerans, Acinetobacter baumannii, and Acinetobacter lwoffii/haemolyticus.*
**Johar et al., 2023**	*Aerococcus spp., Akkermansia muciniphila, Bifidobacterium adolescentis, Bifidobacterium dentium, Clostridioides difficile, Clostridium perfringens, Enterococcus casseliflavus, Enterococcus faecalis, Enterococcus faecium, Lactobacillus gasseri, Lactobacillus johnsonii, Lactobacillus rhamnosus, Streptococcus agalactiae, Escherichia coli, Klebsiella pneumoniae, Morganella morganii, Pseudomonas aeruginosa, Salmonella enterica, Raoultella ornithinolytica, Actinomyces odontolyticus, Propionibacterium acnes, Bacteroides fragilis, Bacteroides vulgatus, Parabacteroides distasonis, Prevotella melaninogenica, Verrucomicrobium spinosum*

[14–28]

Table S. 3 Used Abs panel in all 15 studies.

Author	Antimicrobials Tested
**Eltai et al., 2020**	Ampicillin, Amoxicillin–Clavulanic acid, Tetracycline, Piperacillin-Tazobactam, Colistin, Gentamicin, Amikacin, Ciprofloxacin, Trimethoprim, Sulfamethoxazole, Cephalothin, Cefuroxime, Ceftriaxone, Cefepime, Fosfomycin, Nitrofurantoin, Ertapenem, Meropenem
**Al-Hadidi et al., 2022**	Ampicillin, Amoxicillin/Clavulanic acid, Tetracycline, Piperacillin/Tazobactam, Ciprofloxacin, Trimethoprim/Sulfamethoxazole, Ceftriaxone, Cefepime, Fosfomycin, Nitrofurantoin, Ertapenem, Meropenem
**Johar et al., 2021**	Ampicillin, Cephalothin, Ciprofloxacin, Tetracycline, Fosfomycin, Amoxicillin/Clavulanic Acid, Gentamicin, Cefuroxime, Ceftriaxone, Piperacillin/Tazobactam, Trimethoprim-Sulfamethoxazole, Nitrofurantoin, Colistin
**Al Mana et al., 2022**	Ampicillin, Amoxicillin-Clavulanic Acid, Piperacillin-Tazobactam, Cephalothin, Cefuroxime, Ceftriaxone, Cefepime, Ertapenem, Meropenem, Ciprofloxacin, Tetracycline, Sulfamethoxazole-Trimethoprim, Gentamicin, Amikacin, Fosfomycin, Nitrofurantoin, Colistin.
**Gomez et al., 2021**	Tetracycline, Erythromycin, Penicillin, Streptomycin, Neomycin
**Al-Dulaimi et al., 2019**	Ampicillin, Bacitracin, Cefoperazone, Cephalothin, Clindamycin, Erythromycin, Kanamycin, Novobiocin, Penicillin, Streptomycin, Tetracycline, Vancomycin
**Eltai et al., 2018**	colistin, piperacillin-tazobactam, fosfomycin, ciprofloxacin, nitrofurantoin, amikacin, ampicillin, cephalothin, cefuroxime, ceftriaxone, cefepime, amoxicillin-clavulanic acid, ertapenem, meropenem, trimethoprim-sulfamethoxazole, and tigecycline.
**Eltai et al., 2020a**	ampicillin, amoxicillin-clavulanic acid, piperacillin-tazobactam, amikacin, ciprofloxacin, trimethoprim-sulfamethoxazole, cephalothin, cefuroxime, ceftriaxone, cefepime, colistin, fosfomycin, tigecycline, nitrofurantoin, ertapenem, and meropenem.

**Alhababi et al., 2020**	Penicillins, Ampicillin, Amoxicillin-clavulanic acid, Piperacillin-tazobactam, Cephalothin, Cefuroxime, Ceftriaxone, Cefepime, Ertapenem, Meropenem, Colistin Sulfate, Amikacin, Gentamicin, Tetracycline, Ciprofloxacin, Trimethoprim-sulfamethoxazole, Chloramphenicol, Fosfomycin, Nitrofurantoin
**Islam et al., 2022**	Amikacin, Amoxicillin/Clavulanic Acid, Ampicillin, Cefalotin, Cefovecin, Cefpodoxime, Ceftiofur, Chloramphenicol, Doxycycline, Enrofloxacin, Gentamicin, Imipenem, Nitrofurantoin, Piperacillin, Tetracycline, Tobramycin, and Trimethoprim/Sulfamethoxazole.
**Rahman et al., 2025**	Ampicillin, Amoxicillin-clavulanic acid, Piperacillin-tazobactam, Ertapenem, Meropenem, Amikacin, Gentamicin, Fosfomycin, Trimethoprim-sulfamethoxazole, Ciprofloxacin, Cefotaxime, Ceftazidime, Nitrofurantoin, Tetracycline, Colistin
**Sajjad et al., 2023**	Doxycycline, Clindamycin, Amoxicillin, Metronidazole, Levofloxacin, Ampicillin, Ciprofloxacin, Trimethoprim-Sulfamethoxazole, Amoxicillin-Clavulanate
**Sajjad et al., 2024**	Doxycycline, Cephalexin, Clindamycin, Amoxicillin, Metronidazole, Levofloxacin, Ampicillin, Ciprofloxacin, Trimethoprim/Sulfamethoxazole, Amoxicillin/Clavulanic Acid
**Ibrahim et al., 2024**	Penicillin, Oxacillin, Cefoxitin, Ciprofloxacin, Levofloxacin, Gentamicin, Tobramycin, Amikacin, Erythromycin, Clindamycin, Tetracycline, Trimethoprim-Sulfamethoxazole, Chloramphenicol, Vancomycin, Meropenem, and Colistin.
**Johar et al., 2023**	NA

[14–27]

Table S. 4: Detected ARGs in the included studies.

Author	AMR Genes Detected
**Eltai et al., 2020**	bla_{CTX}*-M* , bla_{TEM} , bla_{SHV}, $bla_{CTX-M-G2}$, *mcr-1*
**Johar et al., 2021**	*ompT, hlyF, iroN, tsh, vat, iss, cvi/cva, iucD*
**Al Mana et al., 2022**	*mcr-1*
**Gomez et al., 2021**	*stx, eae, Bla-*$_{TEM}$, *Bla-*$_{SHV}$, *aadA, AadA*
**Eltai et al., 2018**	*mcr-1*
**Alhababi et al., 2020**	*mcr-1* gene
**Rahman et al., 2025**	*tetA, tetB,* bla_{CTX-M}, *acrS, kpnE, soxR, emrA, acrAB-tolC, acrR, fabI, qacG, marR, mdtE, emrB, AcrE, mdtF, marA*
**Ibrahim et al., 2024**	bla_{CTX}*-M*, bla_{SHV}, bla_{TEM}, *mcr*, bla_{KPC}, bla_{NDM}, bla_{OXA}, bla_{VIM}, bla_{IMP}, *qnr*
**Johar et al., 2023**	bla_{VEB}, bla_{KPC}, bla_{GES}, bla_{VIM-1}, bla_{OXA} variants, *qnrB-1, qnrS, AAc 6′ -1b-cr, tetA, ermB, mefA, aadA1.*

[14,16–18,20,22,24,27,28]).
